# Asthma control in Kazakhstan: need for urgent action

**DOI:** 10.1186/s12890-022-02287-2

**Published:** 2023-01-07

**Authors:** Denis Vinnikov, Aizhan Raushanova, Irina Mukatova, Tair Nurpeissov, Assia Кushekbayeva, Assiya Toxarina, Baktygul Yessimova, Fatima Bespayeva, Nurlan Brimkulov

**Affiliations:** 1 Al-Farabi Kazakh National University, 71 Al-Farabi Avenue, 050040 Almaty, Kazakhstan; 2 Peoples’ Friendship University of Russia (RUDN University), Moscow, Russian Federation; 3 Medial University Astana, Astana, Kazakhstan; 4 Asfendiyarov Kazakh National Medical University, Almaty, Kazakhstan; 5 South-Kazakhstan Medical Academy, Shymkent, Kazakhstan; 6 Regional Clinical Hospital, Shymkent, Kazakhstan; 7 Kyrgyz State Medical Academy, Bishkek, Kyrgyzstan

**Keywords:** Prevalence, Inhaled steroids, Peakflowmetry, GINA

## Abstract

**Background:**

Asthma control, patients’ awareness level and adherence to treatment in Kazakhstan have never been studied. The aim of this study was to verify the prevalence of controlled, poorly controlled and uncontrolled asthma in a large sample of three largest cities of Kazakhstan.

**Methods:**

We recruited 600 (median age 54 (interquartile range (IQR) 22) years, 64% females) patients with diagnosis confirmed earlier in the outpatient facilities in Almaty, Astana and Shymkent in 2020–2021. We offered a structured questionnaire on asthma control and risk factors as of GINA and performed spirometry. We report the prevalence of asthma control, knowledge and skills and pharmacological treatment with 95% confidence interval (CI) and the association of selected predictors with asthma control.

**Results:**

With the median of 9 (IQR 13) years of diagnosis, 40% of patients had comorbid COPD and 42% had allergic rhinitis, whereas 32% lived with pets. Asthma was well-controlled in only 12.3% (95% CI 9.7–15.0), partly controlled in 29.8% (95% CI 26.2–33.5) and uncontrolled in 57.8% (95% CI 53.9–61.8) patients. ACQ-5 score (range 0–5.8, median 2) equaled 0.2 (IQR 0.85) in well-controlled asthma patients, 1.4 (IQR 1) in partly controlled and 2.8 (IQR 1.4) in uncontrolled asthma patients. Knowledge and skills levels were very low. Only 54% were on inhaled corticosteroids (52.2% of them used budesonide/formoterol and 39.5% used fluticasone/salmeterol). 39% used steroids per os or parenterally within a period of 12 months (51% of patients with uncontrolled asthma).

**Conclusion:**

Asthma control, knowledge and skills levels of asthma patients in the largest cities of Kazakhstan remain unacceptably low, whereas pharmacological treatment is far from optimal. Urgent action should be taken to support doctors’ training, and we call to launch a national asthma program to coordinate asthma care in Kazakhstan.

## Background

In Central Asia, including five countries of the former Soviet Union (Kazakhstan, Kyrgyzstan, Tajikistan, Turkmenistan and Uzbekistan), the burden of chronic respiratory diseases (CRD) remains very high, whereas mortality from CRD is one of the highest in the World Health Organization European Region [1]. Economies in transition, high smoking prevalence, very high levels of ambient air pollution, most pronounced in the cold season due to solid fuel combustion for heating [2], occupational exposure to vapors, gas, dust and fumes [3] and the failure of healthcare systems to respond to the growing burden of CRD may explain high prevalence of CRD in the region. Official prevalence data are believed to remain biased, because they are calculated from self-admitted patients and those listed as medication recipients from the state insurance [4]. Few published studies indicate dramatic discrepancy between such data and the findings of the population-based surveys [4, 5].

Chronic asthma is adults and chronic obstructive pulmonary disease (COPD) are the two leading causes of CRD in Kazakhstan, because the detection of other less prevalent diseases, including interstitial, is hampered by poor infrastructure and insufficient vigilance of primary healthcare doctors. Data on COPD prevalence in the epidemiological studies differ dramatically from the official data [4, 5]. Thus, in a study based on forced expiratory flow in one second (FEV_1_)/forced vital capacity (FVC) below lower limit of normality (LLN), COPD prevalence was 8.7% in men and 3.4% in women in the age group 40 years and older [6], which was 10–15 times greater than the official data of the Ministry of Health. Moreover, the epidemiological study was conducted in Almaty, the largest city of Kazakhstan, and the true portrait of COPD prevalence, its control, the use of medications and the contribution of selected risk factor in other parts of the country remain unknown.

As with COPD, very little is known about the true prevalence of asthma in the country, and the only population-based study was completed in Almaty [5], leaving the portrait of asthma elsewhere in the country ulterior. In addition, this study reported doctor-diagnosed asthma and self-reported wheezing, and no verification of asthma diagnosis was performed. Furthermore, no studies of the use of medications, patient’s knowledge and the overall control have ever been published in Kazakhstan. Data of the disease control are partly available from the industry data, because asthma pharmacological treatment is covered by the state insurance in the country. However, selection bias and possible misclassification make these data almost useless for health policy and management. The level of asthma control in Kazakhstan remains unknown, albeit achieving asthma control in the majority of asthma patients is the major goal of treatment (GINA) [7]. We, therefore, conducted this study to verify the prevalence of controlled, poorly controlled and uncontrolled asthma in a large sample of three largest cities of Kazakhstan, including Almaty, Astana and Shymkent.

## Materials and methods

### Study protocol and patients’ referral

The study was planned and implemented in only those patients, who had previous and current history of asthma combined with typical clinical presentation at present, received treatment and were listed as asthma patients in the medical facility registry. In three participating Kazakhstan cities, Almaty, Astana and Shymkent, we recruited patients from the lists of asthma patients, who attended local family medical centers (or polyclinics), adhered to a regular follow-up plan and came to obtain medications from the pharmacies in these family medical centers. In Astana and Shymkent, family doctors also communicated with pulmonologists to organize referral for this study. In addition, in Almaty patients were also referred from the Republican Allergy Center by allergy specialists. Two family medical centers in each city participated in the project. A family doctor, a pulmonologist or an allergy doctor in a polyclinic of Republican Allergy Center invited patients by phone and ask to attend examination in a specially designated room of the Faculty of Healthcare and Medicine of Al-Farabi Kazakh National University (for Almaty), Astana Medical University (for Astana) or the Central Provincial Hospital of Turkestan Province (for Shymkent). We enrolled patients with all types of asthma, including allergic and non-allergic asthma, in May 2020 through January 2021.

Patients were instructed to take their regular medication in the morning, abstain from smoking 12 h prior to the examination, and attend the clinic in the morning to fill in the questionnaire and perform spirometry. We gave patients 30 min to rest after their arrival to allow for shortness of breath to lessen. A qualified internist assisted patients to fill in the questionnaire, which was offered either in Russian (about 90% cases) or Kazakh. Once the questionnaire was filled, we measured patients’ height and weight using electronic calibrated scales. Each patient signed a written informed consent to participate in the study, and the study protocol was approved by the Committee on Bioethics of Al-Farabi Kazakh National University.

### Questionnaire

The questionnaire comprised 53 questions, was a combination or our original questions with standardized tools and included demographic and personal data, questions on medical history, knowledge and skills section, current and former treatment questions and a risk factors section. Demographic and personal data questions were on date of birth, sex, the highest attained education (secondary school, high school, college, university or academic degree), year of first asthma diagnosis, permanent medical disability due to asthma and its group (I, II or II), cigarette smoking status (never-, former or current smoker), exposure to secondhand smoke at home or at work, waterpipe smoking and electronic cigarette use. In those smoking cigarettes, we also asked about the number of cigarettes smoked a day on average and years of smoking. The section on risk factors aimed to be succinct and was only confined to a question on pets at home.

We ascertained asthma control with Asthma Control Questionnaire of five questions (ACQ-5), validated in Russian [8], which allowed to quantify asthma control using a numerical score ranging from 0 to 6. In addition, we also categorized patients into those with well controlled, partially controlled or uncontrolled asthma based on four questions of GINA-2020 guideline. All ‘no’ answers corresponded to well-controlled asthma, one or two ‘yes’ answers to partially controlled asthma and finally 3 or 4 ‘yes’ answers to uncontrolled asthma [7].

Knowledge and skills section covered the questions asking whether a patient knew the address and worktime of the nearest asthma education classes; ever attended that school; attended the school at least once during the preceding year; knew whether peakflowmetry was the major personal self-assessment tool to control asthma and predict exacerbations; whether a patient possessed personal peakflowmeter; performed peakflowmetry twice a day and logged the readings; knew his/her personal peak expiratory flow (PEF) readings corresponding to the green, yellow and red zones; had and maintained asthma diary regularly; and possessed a nebulizer.

Treatment section started with a question whether a patient used any inhaled corticosteroid alone or in combination with a beta-agonist regularly for the last week, and a daily dose. A number of all available brand names on the market were offered in this question. We then asked if a patient used any corticosteroid in pills or injections during the last 12 months. The next question was on a preferred rescue medication and whether a patient has the medication with him/her at all times. we also recorded the number of rescue medication doses during the last 24 h and the last month. We than asked what medication a patient took on a day of examination. This section ended with a question on any other treatments practiced by a patient other than inhaled corticosteroids and beta-agonists.

Risk factors for exacerbations verified in the questionnaire, as listed in GINA [7], included chronic rhinosinusitis, gastroesophageal reflux disease (GERD), confirmed food allergy, pregnancy, allergen exposure, severe exacerbation within the last 12 months, and ever intubation or intensive care for asthma.

### Spirometry

Because the indication for spirometry in this study was to assess treatment effect and disease control, no bronchodilation was performed [7]. Patients arrived to do spirometry after they took their medication, whether inhaled steroids, or bronchodilators, or even aminophylline. We obtained three reproducible maneuvers of vital capacity (VC) and three reproducible maneuvers of forced VC (FVC). The difference between exhalation attempts below 150 ml was indicative of good or acceptable quality. FVC maneuvers lasting at least 6 s, with no end-of-test errors or delayed exhalation, and those with visible maximal muscle attempt during exhalation were considered of acceptable quality and thus were included in the analysis. Both VC and FVC were measured in liters. We also analyzed forced expiratory volume in one second (FEV_1_) in liters and calculated FEV_1_/FVC from the best curve. All three included volumes were reported as actual in liters along with their percent to predicted values. Because predicted values for spirometry in Kazakh populations have never been published, we used Global Lung Function (GLI)-2012 reference equations for European population [6]. In Almaty, we performed all spirometry tests on MAS-2 S (Belintelmed LLC, Belarus). In Astana, we used MicroLoop (CareFusion, United Kingdom) In Shymkent, we chose to work on BTL-08 Spiro (BTL, United Kingdom).

### Statistical analysis

Sample size estimation was guided by the preceding similar studies, but we also considered even more patients with the aim to enroll the maximum number of patients referred from the participating facilities. We tested all continuous variables for normality using Shapiro–Wilk test. Most data were non-normally distributed; therefore, tests used in this study to compare groups were non-parametric, such as Mann–Whitney U test for two-groups comparisons and Kruskall–Wallis test for three or more groups. Groups of binary data were tested for differences using χ2 test from contingency tables, including for three or more groups, such as smoking status of highest attained education. The primary end-point in this analysis was asthma control, expressed as the prevalence of patients with well-controlled, partially controlled and uncontrolled asthma. Prevalence in most groups is expressed as mean with the corresponding 95% confidence interval (CI). Means of normally distributed data are stated as arithmetic means ± standard deviation; alternatively, we report medians with the corresponding interquartile range (IQR). We additionally tested whether such predictors as sex, age, pets at home, comorbid diagnoses, BMI, smoking, years of asthma, exposure to SHS and some other were associated with uncontrolled asthma in bivariate analyses. Because this study mainly planned as descriptive, we did not test predictive role of risk factors in regression models. All tests were completed in NCSS 2020 (Utah, USA), and *p* < 0.05 was a cut-off to consider non-random difference in comparisons.

## Results

### Demographic and lifestyle profile of asthma patients

We enrolled 600 patients with bronchial asthma (36% males) with the median age 54 (IQR 22) years and with the median of 9 (IQR 13) of diagnosis (Table 1). The range of years of asthma was very wide, from 0 to 56 years. Every sixth patient with asthma had disability due to asthma and got compensation. Only 8% of asthma patients smoked cigarettes daily. 40% of patients stated they had ever been told they also had COPD and 42% had allergic rhinitis. One-third of patients lived with pets. Male patients did not differ from their female counterparts in the median age, work duration, years of asthma, BMI, education, exposure to SHS, pets at home; however, there were more daily smokers and fewer never-smokers in men, and their smoking intensity was greater. We also found more waterpipe smokers in men and more patients having comorbid allergic rhinitis in women.

**Table 1:** Demographic and lifestyle profile of included patients

Variable	Overall	Men	Women
N (%)	600 (100)	213 (36)	387 (64)
Age, years	54 (22)	53 (28)	55 (21)
Years worked in ever-workers	24.5 (25)	25 (29.5)	24 (25)
Height, cm	165 (12)	172.8 ± 8.2	161 (8)*
Weight, kg	75 (20)	80.5 ± 13.7	72 (20)*
BMI, kg/m^2^	27.6 (7.3)	26.8 (5.8)	28.0 (7.8)
*Highest attained education, N (%)*			
Secondary school	25 (4)	9 (4)	17 (4)
High school	184 (31)	66 (31)	118 (31)
College or university	388 (65)	135 (64)	251 (65)
Academic degree	3 (0)	2 (1)	1 (0)
Years with diagnosis	9 (13)	9 (12)	9 (14)
Disability, N (%)	89 (15)	36 (17)	53 (14)
Cigarette smoking, N (%)			
Never	403 (67)	92 (43)	311 (80)*
Former	151 (25)	89 (42)	62 (16)
Current	46 (8)	32 (15)	14 (4)
Cig per day in daily smokers	10 (11.3)	10 (12)	6 (7.3)*
Years of smoking in daily smokers	20 (29)	25 (33.3)	20 (16.8)
Exposure to SHS, N (%)	183 (31)	73 (34)	110 (28)
Waterpipe smoking, N (%)	9 (2)	7 (3)	2 (0)*
Electronic cigarette use, N (%)	14 (2)	6 (3)	8 (2)
Pets at home, N (%)	192 (32)	73 (34)	119 (31)
Comorbid COPD, N (%)	241 (40)	92 (43)	149 (39)
Comorbid allergic rhinitis, N (%)	250 (42)	72 (34)	178 (46)*

*BMI *Body mass index; *SHS *Secondhand smoke; *COPD *Chronic obstructive pulmonary disease; means are shown either as medians (interquartile range) or means ± standard deviation

*p < 0.05 from Mann–Whitney U-test for continuous variables or χ2 test for binary variables in the bivariate comparisons (2*3 χ^2^ test for smoking status).

### Asthma control

**Fig. 1:**
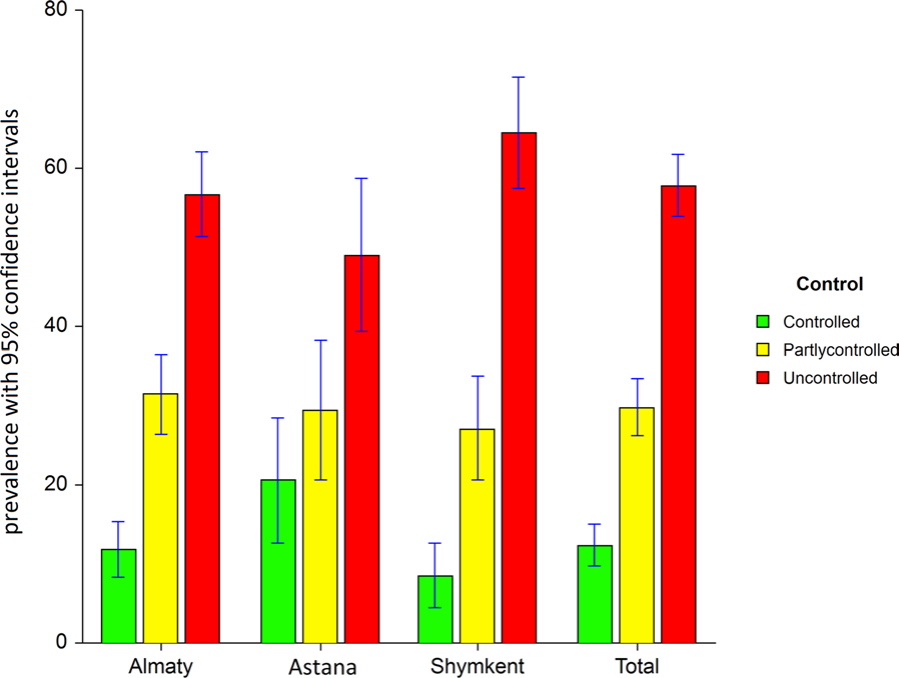
Prevalence of well-controlled, partly controlled and uncontrolled asthma overall and in three cities with the corresponding 95% confidence intervals

Based on four questions of GINA asthma control, asthma was well controlled in only 74 (12.3%; 95% CI 9.7–15.0%), partly controlled in 179 (29.8%; 95% CI 26.2–33.5%) and uncontrolled in 347 (57.8%; 95% CI 53.9–61.8%) patients. When comparing three cities using 2*3 χ2 test, we found significant difference between them (*p* = 0.02) (Fig. 1). There were fewer patients with controlled asthma in Shymkent and more such patients in Astana. Similarly, the prevalence of uncontrolled asthma was the greatest in Shymkent (64.4%; 95% CI 57.4–71.5%), compared to Almaty (56.7%; 95% CI 51.3–62.1%) or Astana (49.0%; 95% CI 39.3–58.7%). Men did not differ from women in the fraction of patients with uncontrolled asthma, 56 and 58%, respectively (*p* = 0.63). In a bivariate analysis, uncontrolled asthma was associated with greater BMI (Mann–Whitney *p* = 0.03), pets at home (64% in those with pets and 55% in those without, χ2 *p* = 0.03) and comorbid COPD (65% vs. 53% in those with no ever-COPD, χ2 *p* < 0.01). The remaining predictors, such as age (Mann–Whitney *p* = 0.14), years of asthma (Mann–Whitney *p* = 0.05), daily smoking (χ2 *p* = 0.44), exposure to SHS (χ2 *p* = 0.96), comorbid allergic rhinitis (χ2 *p* = 0.92) were not associated with uncontrolled asthma.

ACQ-5 score ranged from 0 to 5.8, in which score 2 corresponded to the 50% percentile, score 1.2 was 25th percentile and score 3.0 was 75th percentile, indicative of large fraction of patients with uncontrolled asthma. In well-controlled asthma patients, the median ACQ-5 was 0.2 (IQR 0.85); in partly controlled 1.4 (IQR 1); in uncontrolled asthma patients the corresponding ACQ-5 score was 2.8 (IQR 1.4) (Table 2). Similarly, there was a significant increase in BMI of patients from well-controlled to uncontrolled asthma. In a three-group comparison using Kruskall–Wallis test, we also found significantly poorer lung function: both FEV_1_, FVC and FEV_1_/FVC were lower in patients with uncontrolled asthma. FEV_1_ in a group of uncontrolled asthma was reduced to a median of 67% predicted.

**Table 2:** Demographic, comorbidities and lung function of patients with well-controlled, partially controlled and uncontrolled asthma

Variable	Total	Well controlled	Partly controlled	Uncontrolled
N (%)	600 (100)	74 (12)	179 (30)	347 (58)
Age	54 (22)	50 (29.5)	55 (25)	55 (21)
Years of asthma	9 (13)	7 (10.5)	9 (12)	10 (14.3)
BMI, kg/m^2^	27.6 (7.3)*	26.4 (6.7)	27.3 ± 4.9	27.8 (7.2)
Females, N (%)	387 (64)	45 (61)	116 (65)	226 (65)
ACQ-5 score	2 (1.8)*	0.2 (0.9)	1.4 (1)	2.8 (1.4)
University or academic degree, N (%)	212 (35)*	25 (34)	84 (47)	102 (29)
Comorbid COPD, N (%)	241 (40)*	17 (23)	67 (37)	157 (45)
Comorbid rhinitis, N (%)	250 (42)	31 (42)	74 (41)	145 (42)
FEV_1_, % predicted	76 (34.5)*	84.7 ± 14.6	83 (28)	67 (36.3)
FVC, % predicted	85 (27)*	90.0 ± 15.2	91 (23)	78 (26)
FEV_1_/FVC, %	73 (18)*	79 (9)	75 (14)	68.5 (20.5)

*BMI* Body mass index; *COPD* Chronic obstructive pulmonary disease; *FEV*_1_ Forced expiratory volume in 1 s; *FVC* Forced vital capacity; means are shown either as medians (interquartile range) or means ± standard deviation

*p < 0.05 in a three-group comparison either from Kruskall–Wallis test (continuous data) or from 2*3 χ^2^ test (binary data)

### Knowledge and skills

Only 3% of asthma patients in Kazakhstan knew the location and work schedule of the nearest asthma education classes; 7% ever attended class in them; 2% attended at least one training session within the last year; 10% knew that peakflowmetry was a preferred way to monitor asthma at home; 4% had a peakflowmeter at home; 3% knew exact peak expiratory flow (PEF) readings for their green, yellow and red zones; only 1% performed peakflometry at home daily; and only 4% had a diary of symptoms. 54% of patients possessed a personal nebulizer at home, but that did not affect asthma control (50% of patients with well-controlled asthma; 51% with partly controlled and 56% with uncontrolled ailment).

### Pharmacological treatment

**Fig. 2:**
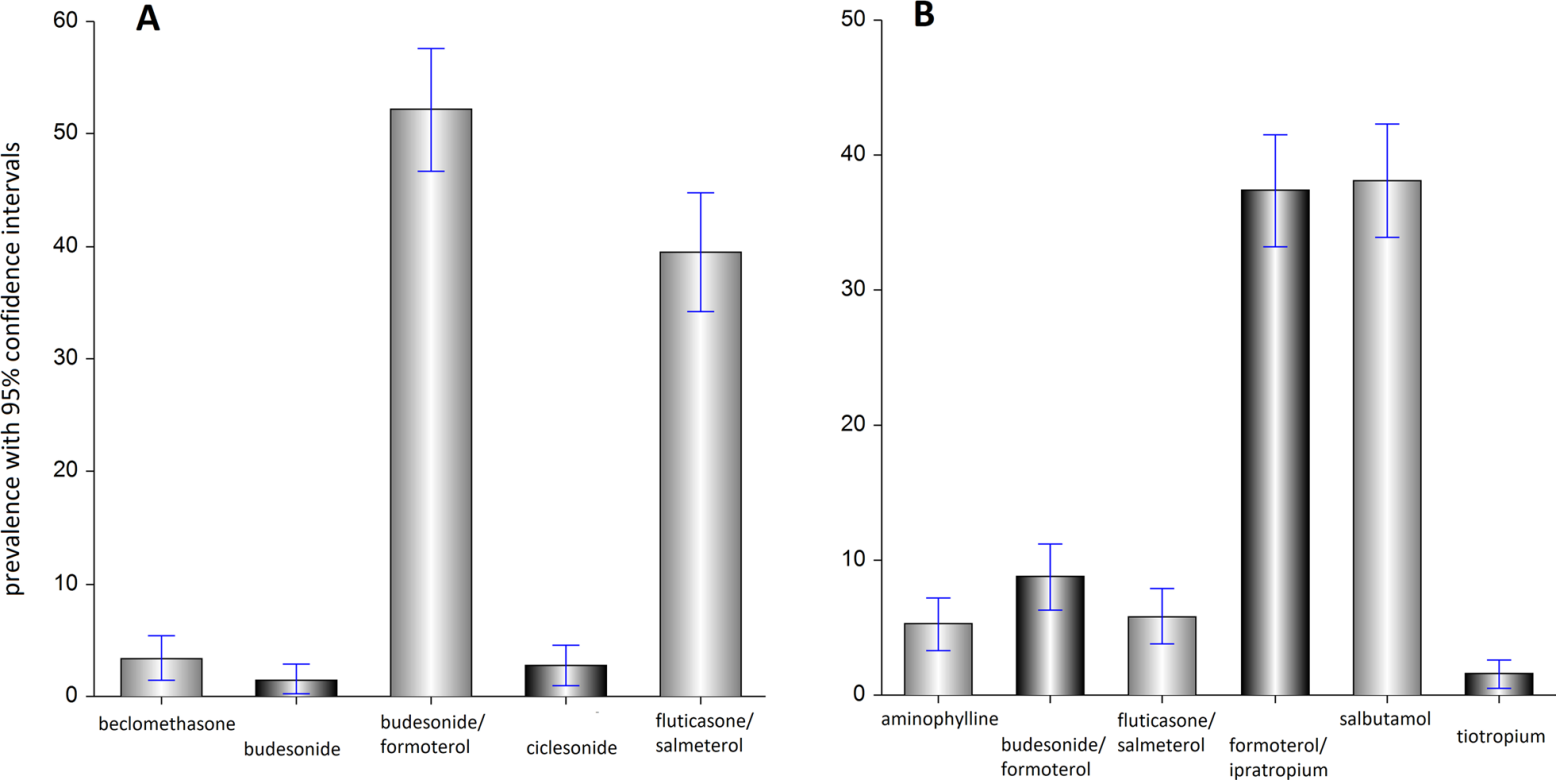
The structure of medications used as basic treatment (**A**) and as rescue medications (**B**)

Only half of the group (N = 324; 54%) were on inhaled corticosteroids alone or in combination with a long-active beta-agonist a week prior to examination. The fraction of inhaled steroids users significantly increased from 31% in a group of well-controlled asthma to 62% in uncontrolled asthma (Table 2). In the latter, 51% stated they used peroral or parenteral steroids within a 12-months period prior to examination, indicative of very poor compliance with asthma treatment recommendations, resulting in no or low effect of inhaled steroids, which is in turn consistent with very low knowledge level and patients’ training coverage. Of 324 patients using inhaled steroids regularly, most patients (52.2%) used budesonide/formoterol; followed by 39.5% using fluticasone/salmeterol; 3.4% beclomethasone; 2.8% ciclesonide; 1.5% budesonide; and fewer than 1% used fluticasone or vilanterol/fluticasone (Fig. 2A). In case of acute attack or shortness of breath (Fig. 2B), the range of rescue medications preferred by patients included salbutamol (38.1%); fenoterol/ipratropium (37.4%); budesonide/formoterol (8.8%); fluticasone/salmeterol (5.8%); aminophylline pills or injections (5.3%); tiotropium (1.6%); and beclomethasone (1.0%). Ciclesonide, fenoterol, indacaterol, pills of dexamethasone or prednisolone were used by less than 1% patients for this purpose.

Patients receiving inhaled steroids regularly had generally more severe disease. Thus, their ACQ-5 score was greater (median 2.4 vs. 1.8, Mann–Whitney *p* < 0.001) with worse lung function. Their median FEV_1_% was 71% versus 82% predicted (Mann–Whitney *p* < 0.001), median FVC 80.5% versus 88% predicted (Mann–Whitney *p* < 0.01) and median FEV_1_/FVC 70% versus 77% (Mann–Whitney *p* < 0.001).

### Risk factors

Chronic rhinosinusitis was present in 137 (23%) patients (Table 3) and was associated with greater ACQ-5 score (median 2.4 vs. 2, *p* < 0.05); GERD was reported by 72 (12%) patients; confirmed food allergy was found in 100 (17%) and exposure to allergens in 214 (36%). Severe asthma exacerbation during the preceding 12 months was reported by 262 (44%) patients and was associated with greater ACQ-5 score (median 2.8 vs. 1.6, p < 0.001), lower FEV_1_% predicted (median 67.5 vs. 81%, *p* < 0.001), FVC% predicted (median 79.5 vs. 87, *p* < 0.001) and even FEV_1_/FVC (median 68.5 vs. 75%, *p* < 0.001). Twelve percent (N = 71) were ever-intubated or treated in an intensive care unit, and that predicted worse ACQ-5 score (median 2.8 vs. 2.1, *p* < 0.001), lower FEV_1_% predicted (median 61.2 vs. 75.4%, *p* < 0.001), FVC% predicted (median 76.6 vs. 84.2, *p* < 0.001) and even FEV_1_/FVC (median 64.9 vs. 71.7%, *p* < 0.001).

**Table 3:** Knowledge, skills, risk factors and pharmacological treatment in the studied sample

Variable	Total	Well controlled	Partly controlled	Uncontrolled
N (%)	600 (100)	74 (12)	179 (30)	347 (58)
*Knowledge and skills*				
Know the location and work schedule of the nearest asthma education classes	18 (3)*	0 (0)	11 (6)	7 (2)
Ever attended classes	41 (7)	2 (3)	12 (7)	27 (8)
Attended at least one training session within the last year	13 (2)	3 (4)	5 (3)	5 (1)
Know that peakflowmetry was a preferred way to monitor asthma at home	59 (10)	8 (11)	19 (11)	32 (9)
Have a peakflowmeter at home	21 (4)	3 (4)	8 (5)	10 (3)
Know exact PEF readings for their green, yellow and red zones	19 (3)	2 (3)	7 (4)	10 (3)
Perform peakflometry at home daily	3 (1)	1 (1)	2 (1)	0 (0)
Have a diary of symptoms	21 (4)	3 (4)	3 (2)	15 (4)
*Risk factors*				
Chronic rhinosinusitis	137 (23)	16 (22)	32 (18)	89 (26)
GERD	72 (12)	4 (5)	20 (11)	48 (14)
Confirmed food allergy	100 (17)*	10 (14)	18 (10)	72 (21)
Pregnancy	4 (1)	2 (3)	1 (1)	1 (0)
Exposure to allergens	214 (36)*	18 (24)	51 (28)	145 (42)
Severe asthma exacerbation during the preceding 12 months	262 (44)*	10 (14)	50 (28)	202 (58)
Ever intubated or in intensive care unit for asthma	71 (12)*	4 (5)	11 (6)	56 (16)
Psychological problems	90 (15)*	7 (9)	18 (10)	65 (19)
Pets at home	192 (32)	21 (28)	48 (27)	123 (35)
*Pharmacological treatment*				
Daily use of inhaled corticosteroids	324 (54)*	23 (31)	85 (47)	216 (62)
Use of steroids per os or parenterally in the last 12 months	233 (39)*	9 (12)	46 (26)	178 (51)

Data era presented as N (%);* PEF* Peak expiratory flow;* GERD* Gastroesophageal reflux disease

* p < 0.05 in a three-group comparison from 2*3 χ^2^ test

## Discussion

This is the first report of asthma control in patients residing in three major cities of Kazakhstan, including the capital, summarizing the data of 600 patients. We found that only every sixth asthma patient in urban Kazakhstan had his/her asthma under full control, whereas the fraction of patients with uncontrolled asthma was unacceptably large with some variation between the cities. Control skills and knowledge level of patients was very low, only half of patients were on regular inhaled steroids treatment and almost 40% used steroids *per os* or parenterally within one year. Taken together, our data draw a picture of unacceptably low level of asthma control in three largest cities of Kazakhstan, despite the fact that asthma pharmacological treatment, including combined inhaled steroids with long-acting bronchodilators, is covered by the state.

Prior to this presentation, the portrait of asthma control in the region of Central Asia remained unknown. Our literature search could not detect a scientific summary of asthma control to-date in any country of the region, including Kazakhstan, Kyrgyzstan, Uzbekistan, Tajikistan and Turkmenistan. In the Russian Federation, few published studies showed that a great number of asthma patients unlikely have their asthma controlled. One of recent published studies demonstrated that the prevalence of uncontrolled asthma using GINA tool of asthma control was much greater compared to self-reported asthma in a study of 259 patients (3%, but no breakdown to countries studied) [9]. In addition, no spirometry was performed to verify asthma or its control. The magnitude of estimates of asthma control elsewhere is so large, that contrasting results are usually reported from the same country. Up to 64% asthma cases were uncontrolled in Poland [10], up to 52% in Turkey [11], but up to 34–37% totally controlled in another study [12]. The fraction of uncontrolled asthma was 35% in women in Finland [13]. But can be as low as 10% in Czech Republic [14], 24% in Greece [15], 30% in Romania [16], 13% in Hungary [17], 13% in Sweden [18], with good and optimal control in 67% in a nation-wide study in Italy [19]. Many studies listed above did not verify self-reported asthma control with spirometry.

Insufficient asthma control in Kazakhstan presented in this study is likely the outcome of poor training and unacceptable follow-up of patients. Asthma patients get admission to the outpatient facilities along with all other numerous reasons of admission for other patients, thus find themselves in a situation of very limited time and almost no opportunities to be trained how to use an inhaler, how to monitor asthma, what to do in case of symptom worsening, etc. Very poor awareness level on asthma symptoms management, use of inhalers and the value of peakflowmetry in our patients result from such improper time management of a general practitioner. However, very limited asthma knowledge was earlier reported elsewhere, such as in Turkey [11]. With a due approach to timing and, hence, closer and trustworthy contact with a patient, primary care nurses and doctors could allocate more time to train how to use inhalers and control the disease. Examples of excellent patient’s knowledge and skills exist, such as in Serbia, where patients were already well-trained in the beginning of a program, but showed even further statistically significant skills levels on the program completion [20]. Data on the compliance of patients with treatment, such as the use of inhalers, however, have never been published in Kazakhstan or four neighboring countries of Central Asia.

One of surprising findings of this study was a frequent use of formoterol/ipratropium combination for asthma attack relief. The rationale for highly prevalent ipratropium use in asthma patients in Kazakhstan lies in the axis of medications procurement by the state medical facilities. Pharmacological treatment of both asthma and COPD patients listed as chronic patients is free of charge for patients and covered by the state programs. Therefore, buying one rescue medication instead of a range of other options for both patient groups saves time for personnel responsible for procurement. High prevalence of peroral corticosteroids and aminophylline is explained by inability to attain asthma control with inhaled steroids resulting from a combination of reasons (poor awareness levels, no training, low motivation of a general practitioner, etc.) [6]. Peroral steroids and aminophylline are easy for a patient to use, do not require training and yield immediate effect. Local protocol does not reserve these medications for asthma treatment, yet these are preferred by selected patients, but such practice should be terminated, once proper and sufficient training becomes available.

Poor asthma control and very low skills and awareness levels of asthma patients necessitate further speculation [6]. We believe that scarce availability of specialized care such as access to pulmonologists in most parts of the country, even including the largest cities; limited access to high-quality spirometry and its underrating for asthma control; low motivation of nurses in the outpatient facility to allocate time to work with asthma patients due to low salaries; no budget for asthma education classes in the local outpatient facilities, resulting in no asthma education classes at present in all included cities; and finally the absence of national coordinating body and a national program for asthma control explain meager asthma control in Kazakhstan in this study. In addition, current pulmonologists’ training curricula and state order cannot guarantee sufficient knowledge and skills of a practitioner in the future practice. Of note, none of these components of care have ever been scientifically assessed and discussed in Kazakhstan.

We consider inclusion of all three major cities with a population close to or more than one million people in Kazakhstan a strength of this presentation. The use of both GINA asthma control and risk factors questionnaire with ACQ-5 test, supplemented by spirometry is also an advantage of our study. Nevertheless, our study was limited to urban population only, whereas rural population may have even worse asthma control due to limited access to learning resources, direct access to medical facilities and medications [6]. Therefore, our findings of very high prevalence of uncontrolled asthma may be further underestimated when the entire population of the country is considered. Besides, we designed our study to recruit patients who are listed on the patients’ lists in the outpatient medical facilities as those with chronic asthma and receiving treatment; therefore, patients with very mild and yet undiagnosed asthma may have escaped from this study. In addition, patients’ enrollment was extended from spring to winter next year because of logistic obstacles and thus asthma control may have been affected by the season, which we did not consider in data analysis.

In conclusion, this first report of asthma control in Central Asia demonstrated that yet very low fraction of adult asthma patients has their asthma under control, knowledge and skills of asthma patients are unacceptably poor, whereas pharmacological treatment is far from optimal and is not always consistent with GINA recommendations. Given that asthma pharmacological treatment in Kazakhstan is paid by the state, effort should be made to attain better asthma control with more resources allocated to doctors’ and patients’ training, improvement in spirometry quality and access to it, as well as sustainable research in asthma care, including the countryside. These findings might be more generalizable to the country’s urban areas and that more research is needed to understand the prevalence of asthma control in the rural regions of the country.

## Data Availability

All data generated or analysed during this study are included in this published article.

## Abbreviations

ACQ-5: Asthma control questionnanire-5
BMI: body mass index
CI: confidence interval
COPD: chronic obstructive pulmonary disease
CRD: chronic respiratory diseases
FEV_1_: forced expiratory volume in one second
FVC: forced vital capacity
GERD: gastroesophageal reflux disease
GINA: Global Initiative for Asthma
GLI: Global Lung Function Initiative
IQR: interquartile range
LLN: lower limit of normality
PEF: peak expiratory flow
SHS: secondhand smoke
